# Internal Limiting Membrane Peeling and Flap Inverting under Air in Large Idiopathic Macular Hole Surgery

**DOI:** 10.1155/2021/2003001

**Published:** 2021-09-24

**Authors:** Yuan Zong, Kaicheng Wu, Jian Yu, Changbo Zhou, Chunhui Jiang

**Affiliations:** ^1^Department of Ophthalmology and Vision Science, Eye and ENT Hospital, Fudan University, Shanghai 200031, China; ^2^Key Laboratory of Myopia of State Health Ministry, Key Laboratory of Visual Impairment and Restoration of Shanghai, Shanghai 200031, China; ^3^Department of Ophthalmology, Zhejiang Putuo Hospital, Zhoushan, Zhejiang Province, China

## Abstract

**Purpose:**

To evaluate the efficacy of internal limiting membrane (ILM) peeling combined with modified flap inverting under air in the treatment of large idiopathic macular hole (MH).

**Methods:**

Eyes with a large idiopathic MH (minimum diameter >550 *μ*m) were included in this study. The surgical procedure included standard 23-gauge pars plana vitrectomy (PPV), ILM peeling, complete fluid-gas exchange, and ILM flap inversion under air. The patients underwent follow-up exam including optical coherence tomography (OCT) and best-corrected visual acuity (BCVA) measurement.

**Results:**

Sixteen eyes from 16 patients were included. Mean MH diameter was 681.43 ± 112.12 *μ*m. After a mean follow-up time of 6.25 ± 2.65 months, in all cases, the MH was closed, and the ILM flap could be seen at the inner surface of the fovea. U-shaped and V-shaped MH closure was achieved in 11 and 5 cases, respectively. The BCVA improved significantly from 1.49 ± 0.35 logMAR to 0.89 ± 0.35 logMAR (*p* < 0.05), and visual acuity of 20/100 or better was achieved in 8 eyes.

**Conclusion:**

ILM flap inverting under air was helpful in improving the functional and anatomic outcomes of vitrectomy for large idiopathic MH.

## 1. Introduction

Idiopathic macular hole (MH) is a common finding in the clinic, with an incidence of about 8.5 individuals per 100,000 population per year [1]. In 1991, Kelly and Wendel [2] first reported the treatment of MH using pars plana vitrectomy (PPV). Although the primary result was encouraging, 2%–32% of the MH closure cases remained unclosed [2–4]. Many other methods have been tried to improve the outcome [5, 6]. In 2010, Michalewska et al. [7] introduced the inverted internal limiting membrane (ILM) flap technique, which greatly improved the postoperative closure rate for large MH and has been widely used since then. This technique has certain drawbacks such as easy detachment of the ILM flap from the edge of the MH during fluid-air exchange [8]. Many researchers have devoted their time to modify this technique to overcome these limitations [9, 10]. Herein, we describe a modified method, manipulating the ILM flap under air; furthermore, the surgical outcomes of this modified technique were evaluated.

## 2. Methods

This retrospective study was approved by the Ethics Board of the Eye, Ear, Nose, and Throat (EENT) Hospital, Fudan University, and the procedures adhered to the tenets of the Declaration of Helsinki. All participants had given their written informed consent. Patients who were diagnosed with a large idiopathic MH with a diameter of >550 *μ*m [11] and treated by a single surgeon (CH. J) at the EENT Hospital between April 2019 and January 2020 were recruited. The inclusion criteria were as follows: clinical presentation of MH with a diameter of >550 *μ*m, without retinal detachment; intraocular pressure of <21 mmHg, axial length (AL) between 21 and 25 mm, and SE between +1 and −3 diopter (*D*). Patients with a history of proliferative vitreoretinopathy, intraocular diseases other than cataract, and those who underwent retinal surgery previously were excluded. Data of the general medical history and ophthalmic history were carefully recorded. Standard eye examinations were performed before surgery and at each visit after surgery, including best-corrected visual acuity (BCVA), slit-lamp biomicroscopy examination, and dilated fundus examination using a slit-lamp with an 84 *D* lens. Axial length (AL) was measured before surgery using an optical biometer (IOLMaster, version 3.01; Carl Zeiss Meditec, Jena, Germany), and intraocular pressure (IOP) was determined using a noncontact tonometer (Canon TX-20 full auto tonometer, Canon, Japan).

Intraoperative and postoperative complications were recorded. Optical coherence tomography (OCT) (using SPECTRALIS HRA OCT; Heidelberg Engineering, Heidelberg, Germany) was performed before and at 1 week and 1, 3, 6, and 9 months after surgery. MH diameter was defined as the minimum opening diameter as measured on horizontal OCT scans across the center [12]. Macular closure type was defined according to the classification detailed by Imai et al. [13]: U-shaped closure is similar to that of the normal macula that has a smooth circular surface. V-shaped closure is a steep foveal contour that has a notch where the neurosensory layer is extremely thin. W-shaped closure has the foveal defect of the neurosensory retina with flattened cuff.

### 2.1. Surgical Technique

All surgeries were performed by a single retina specialist (CH. J) under a retrobulbar block. The pupil was dilated with a combination of phenylephrine 5% and tropicamide 1%. The cataract was removed, and the posterior capsule was left intact. Then, standard 3-port 23-gauge PPV was performed with a noncontact wide-angle viewing system. The Resight 500 was used in 14 cases, and the Oculus BIOM^®^ 5 system was used in 2 cases. Vitrectomy was first performed, followed by posterior hyaloid removal with the help of triamcinolone acetonide. After that, the ILM was stained with indocyanine green (ICG, 2.5 mg/mL, Dandong Yichang Pharmaceutical Co., China), and the ILM was peeled starting from the inferior vascular arcade. The peeling was stopped at a distance about 50–100 *μ*m from the edge of the MH. After the peeling of the 360° ILM, the ILM flap was trimmed using a vitreous cutter, and the central part (about 1.5 papillary diameter) was left attached to the edge of the hole. Then, complete fluid-gas exchange was performed, and the air pressure lowered to about 20 mmHg. The ILM flap was then inverted. Several attempts were occasionally required before the flap was picked and inverted (ILM flap inverted step under air in two cases, https://drive.google.com/file/d/1MEKTMQQM4J_TAYAr7iA51f6Oi_mm59rJ/view?usp=sharing). The reflex from the interface between air and fluid would change when the ILM forceps reached the interface; sometimes, this could be used as a sign for the moment to pick the ILM flap. The ILM flap could be inverted over the MH in two or more directions. If there was too much balanced salt solution (BSS) over the posterior pole, then the BSS was removed using a backflush needle. And in case, the flap moved from the ideal place with the removal of BSS, the procedure of ILM inverting was performed again. The trocars were then removed. Patients were introduced to have a facedown positioning for 7 days postoperatively.

### 2.2. Statistics

All analyses were performed using SPSS software, version 16.0 (SPSS Inc., Chicago, USA). The level of significance was set at *p* < 0.05. Data are presented as the mean ± standard deviation (SD). Student's *t*-test was used to compare the preoperation and postoperation data.

## 3. Results

Sixteen eyes from 16 patients with a large idiopathic MH were included in this study. Patients' demographic data are given in Table 1. Mean symptom duration was 29.06 ± 23.24 months, and mean AL was 23.17 ± 0.84 mm. The mean diameter of the MH was 681.44 ± 112.12 *μ*m. In case 5, a very tiny retinal hemorrhage was noticed at the place where the ILM was picked. No other intraoperative or postoperative complications were noted.

**Figure 1:**
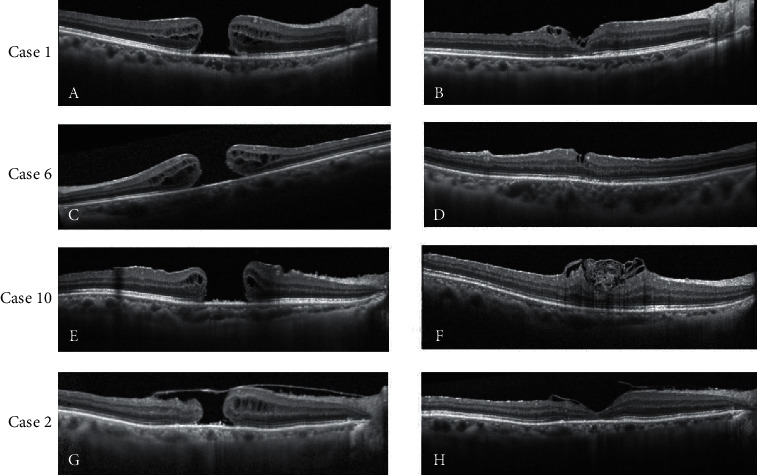
Preoperative and postoperative optical coherence tomographic (OCT) scan images of four cases. (a-b) Preoperative and postoperative OCT scan images of case 1. (c-d) Scan images of case 6. (e-f) Scan images of case 10. (g-h) Scan images of case 2 before and after the second procedure.

In 15 cases, the MH closure was achieved after primary surgery (Figures 1(a)–1(f)). While in case 2, with the ILM over the MH (Figure 1(g)), the latter remained open after the first operation. The patient received a second fluid-air exchange and ILM manipulation under air. A U-shaped closure was achieved 3 months later (Figure 1(h)). After a mean follow-up duration of 6.25 ± 2.65 months, the BCVA improved significantly from 1.49 ± 0.35 logMAR to 0.89 ± 0.35 logMAR (*p* < 0.05); visual acuity of 20/100 or better was achieved in 8 eyes. In all cases, the MH was closed, and the ILM could be seen at the inner surface of the retina at the fovea. In 11 cases, a U-shaped MH closure was achieved, and in the other 5 cases, a V-shaped MH closure was achieved (Figure 1).

## 4. Discussion

Using a modified ILM flap inverting technique, large idiopathic MHs were successfully treated with PPV and air tamponade. Postsurgery OCT examination confirmed that, in all cases, the MH was closed and the ILM was lying over the central fovea.

With PPV, ILM peeling, and gas tamponade, most of the idiopathic MH cases could be successfully treated. For refractory cases, ILM flap inverting has been developed, but always with expansible gas or silicone and face down position. Even so, closure rate was not 100% [14]. Michalewska et al. [7] suggested the inverted ILM could be regarded as a scaffold for tissue proliferation and may provide an environment in which photoreceptors can assume new positions in direct proximity to the fovea. Also, the ILM contains Müller cell fragments. However, in some cases, MH closure was still not achieved, possibly because the ILM flap detached spontaneously after the fluid-air exchange [7]. To solve this problem, here we reported a modification of ILM peeling and flap inverting technique—invert ILM flap under air. During follow-up, in all cases, MH closure was achieved and the ILM flap could be seen at the inner surface of the fovea.

On the other hand, for special MHs, including large, persistent, and highly myopic, special techniques were developed such as perfluorocarbon liquid-assisted inverted limiting membrane flap technique [15], internal limiting membrane transposition and tuck technique [16], inverted ILM flap combined autologous blood clot technique [17], the neurosensory retinal flap [18], human amniotic membrane plug transplant [19, 20], and lens capsular flap transplantation [21]. These techniques achieved a high success rate, but limitations such as retinal toxicity and risk of infection were also noticed (Supplementary Table). The modification we presented this time has certain advantages. First, one can be sure about the position of the ILM flap lying over the MH at the end of the surgery. During follow-up visits, the ILM was found above the fovea in all the cases. In case 2, although the MH remained open, the ILM was seen over the MH. It is worth mentioning that although there are several ILM layers seen in case 11 postoperation, the inverted ILM flap technique was used in our study which was different from ILM insertion. The neurosensory retina showed a complete recovery in case 11. While in former studies, ILM insertion or inverted over the hole had different outcomes on outer nuclear layers, even if the hole closes [22].

Second, this modification is simple, straightforward, and does not require any additional procedure, instrument, or material, making it more economical and greatly reducing the risk of infection and retinal toxicity. Furthermore, in cases where the hole persists, one can perform fluid-air exchange and repeat the procedure again, as we detailed in case 2. Moreover, during the study period, long-term gas was not available in China; hence, only air was used in our cases. Thus, with this modification, the prone positioning time, which was required after MH surgery, could be reduced remarkably. Also, the large macular hole closed in all cases. Compared to other studies [11, 14], our patients had a longer average duration of disease (average 29.06 ± 23.24 months) and the baseline vision was poor (1.49 ± 0.35 logMAR); however, they had a mean 0.6 logMAR improvement in BCVA, which was comparable to or even better than that reported in other studies (Supplementary Table).

Some key points should be noted about the procedure. The first point is to provide a clear view of the posterior pole. The posterior capsule must be left intact and kept moist. With a dried posterior capsule or posterior IOL surface, the view would be extremely poor and manipulation of the ILM flap under air would be near-impossible. In all our cases, the wide-angle viewing system was used, and the author found it difficult to perform this technique with a flat contact lens. The second point is the control of BSS over the posterior pole. The reflex from the interface between air and fluid would change when the ILM forceps touches the interface. This could be a sign for when to pick the ILM flap with the ILM forceps. With too much BSS, this sign does not work well. With excess BSS, the ILM might not stay stable at the place it was left. Using a backflush needle, the extra BSS should be removed. Usually, after a thorough fluid-air exchange, within a few seconds, some BSS would be accumulated over the area, and this would be the optimal moment to pick the ILM flap. Furthermore, as only a very tiny part of the ILM was left connected to the retina underneath, the air pressure was lowered to around 20 mmHg when the remaining fluid at the posterior pole was cleaned. With high air pressure, the ILM flap might accidentally disappear into the backflush needle with the fluid. One might notice the hemorrhage at the macular area in https://drive.google.com/file/d/1MEKTMQQM4J_TAYAr7iA51f6Oi_mm59rJ/view?usp=sharing, and this was a result of the low intraocular pressure. Iatrogenic damage to the retina might be of concern; in only one of our initial cases, a very tiny retinal hemorrhage was noticed at the place the ILM was picked. In this study, we presented the results of our primary cases, and we have used this modification in more than 20 cases; no other intra or postoperative complications were noticed.

However, our cases were limited, and we tried this surgery in highly myopic cases, but the posterior staphyloma made the visualization more challenging; the efficacy of this surgery in highly myopic cases or other macular surgery needs to be studied in the future.

With our primary results, ILM flap inverting under air was helpful in improving the functional and anatomic outcomes of vitrectomy for large idiopathic MH.

## Figures and Tables

**Table 1:** Demographics of patients with a large macular hole.

Patient no.	Age (years)	Gender	Laterality	AL (mm)	MH diameter (*μ*m)	BCVA (Snellen)		BCVA (logMAR)		Follow-up (months)	Type of hole closure
						Initial	Final	Initial	Final		
1	64	F	Right	23.10	618	20/200	20/100	1.0	0.7	7	U
2	72	F	Right	23.64	600	20/400	20/100	1.3	0.7	3	U
3	59	F	Right	23.29	603	20/1000	20/200	1.7	1.0	5	V
4	65	F	Right	23.69	696	20/2000	20/133	2.0	0.8	8	V
5	76	F	Left	22.08	914	20/1000	20/1000	1.7	1.7	8	V
6	67	F	Left	22.32	609	20/1000	20/400	1.7	1.3	9	U
7	68	F	Right	22.30	669	20/1000	20/100	1.7	0.7	10	V
8	65	F	Right	21.96	574	20/200	20/50	1.0	0.4	4	U
9	69	M	Left	22.90	627	20/200	20/67	1.0	0.52	10	U
10	49	F	Right	22.54	920	20/400	20/200	1.3	1.0	4	U
11	69	F	Left	23.33	716	20/400	20/200	1.3	1.0	3	U
12	67	M	Right	23.67	550	20/667	20/100	1.5	0.7	10	U
13	71	F	Right	24.06	626	20/400	20/67	1.3	0.51	6	U
14	48	F	Right	24.56	796	20/2000	20/333	2	1.22	6	V
15	70	F	Left	22.67	746	20/400	20/100	1.3	0.7	3	U
16	57	F	Left	24.67	639	20/2000	20/400	2	1.3	4	U

AL, axial length; MH, macular hole; BCVA, best-corrected visual acuity.

## Data Availability

The data used to support the findings of this study are available from the corresponding author upon request.

## Supplementary Materials

Supplementary Materials Supplementary Table. Comparison of special techniques used for refractory macular holes.

## References

[1] McCannel C. A., Ensminger J. L., Diehl N. N., Hodge D. N. (2009). Population-based incidence of macular holes. *Ophthalmology*.

[2] Kelly N. E., Wendel R. T. (1991). Vitreous surgery for idiopathic macular holes. *Archives of Ophthalmology*.

[3] Haritoglou C., Gandorfer A., Kampik A., Tognetto D. (2004). Anatomic and visual outcomes after indocyanine green-assisted peeling of the retinal internal limiting membrane in idiopathic macular hole surgery. *American Journal of Ophthalmology*.

[4] Beutel J., Dahmen G., Ziegler A., Hoerauf H. (2007). Internal limiting membrane peeling with indocyanine green or trypan blue in macular hole surgery. *Archives of Ophthalmology*.

[5] Thompson J. T., Glaser B. M., Sjaarda R. N., Murphy R. P., Hanham A. (1994). Effects of intraocular bubble duration in the treatment of macular holes by vitrectomy and transforming growth factor-beta 2. *Ophthalmology*.

[6] Olsen T. W., Sternberg P., Capone A. (1998). Macular hole surgery using thrombin-activated fibrinogen and selective removal of the internal limiting membrane. *Retina*.

[7] Michalewska Z., Michalewski J., Adelman R. A., Nawrocki J. (2010). Inverted internal limiting membrane flap technique for large macular holes. *Ophthalmology*.

[8] Chatziralli I., Machairoudia G., Kazantzis D., Theodossiadis G., Theodossiadis P. (2021). Inverted internal limiting membrane flap technique for myopic macular hole: a meta-analysis. *Survey of Ophthalmology*.

[9] Hirata Y., Yuda K., Odontuya D., Hayashi T., Suzuki Y. (2019). A viscoelastic aspiration technique for autologous transplantation of the free-flap inner limiting membrane during macular hole surgery. *Retina*.

[10] Shin M. K., Park K. H., Park S. W., Byon I. S., Lee J. E. (2014). Perfluoro-n-octane-assisted single-layered inverted internal limiting membrane flap technique for macular hole surgery. *Retina*.

[11] Yamashita T., Sakamoto T., Terasaki H. (2018). Best surgical technique and outcomes for large macular holes: retrospective multicentre study in Japan. *Acta Ophthalmologica*.

[12] Ullrich S., Haritoglou C., Gass C., Schaumberger M., Ulbig M. W., Kampik A. (2002). Macular hole size as a prognostic factor in macular hole surgery. *British Journal of Ophthalmology*.

[13] Imai M., Iijima H., Gotoh T., Tsukahara S. (1999). Optical coherence tomography of successfully repaired idiopathic macular holes. *American Journal of Ophthalmology*.

[14] Park J. H., Lee S. M., Park S. W., Lee J. E., Byon I. S. (2019). Comparative analysis of large macular hole surgery using an internal limiting membrane insertion versus inverted flap technique. *British Journal of Ophthalmology*.

[15] Hu Z., Gu X., Qian H. (2019). Perfluorocarbon liquid-assisted inverted limiting membrane flap technique combined with subretinal fluid drainage for macular hole retinal detachment in highly myopic eyes. *Retina*.

[16] Fung N. S. K., Mak A. K. H., Yiu R., Wong I. Y. H., Lam W. C. (2020). Treatment of large, chronic and persistent macular hole with internal limiting membrane transposition and tuck technique. *International Journal of Retina and Vitreous*.

[17] Hu Z., Lin H., Liang Q., Wu R. (2020). Comparing the inverted internal limiting membrane flap with autologous blood technique to internal limiting membrane insertion for the repair of refractory macular hole. *International Ophthalmology*.

[18] Grewal D. S., Mahmoud T. H. (2016). Autologous neurosensory retinal free flap for closure of refractory myopic macular holes. *JAMA Ophthalmology*.

[19] Rizzo S., Caporossi T., Tartaro R. (2019). A human amniotic membrane plug to promote retinal breaks repair and recurrent macular hole closure. *Retina*.

[20] Ferreira M. A., Maia A., Machado A. J. (2021). Human amniotic membrane for the treatment of large and refractory macular holes: a retrospective, multicentric, interventional study. *International Journal of Retina and Vitreous*.

[21] Chen S.-N., Yang C.-M. (2016). Lens capsular flap transplantation in the management of refractory macular hole from multiple etiologies. *Retina*.

[22] Faria M. Y., Proença H., Ferreira N. G., Sousa D. C., Neto E., Marques-Neves C. (2020). Inverted internal limiting membrane flap techniques and outer retinal layer structures. *Retina*.

