# Supplementary material for: Internal Limiting Membrane Peeling and Flap Inverting under Air in Large Idiopathic Macular Hole Surgery
Source: J Ophthalmol. 2021 Sep 24;2021:2003001. doi: 10.1155/2021/2003001 (PMC8487370; doi:10.1155/2021/2003001)
Supplement: Supplementary Materials — Supplementary Table. Comparison of special techniques used for refractory macular holes. [file 2003001.f1.docx]

Supplementary Table. Comparison of special techniques used for refractory macular holes

| Author | Technique | MH diameter | Improved BCVA log MAR | Closure rate | Case number | Temponade | Limitation |
| --- | --- | --- | --- | --- | --- | --- | --- |
| Hu Z, et al. | Perfluorocarbon Liquid-Assisted ILM Flap Technique | No statement | 1.22 | 100% | 13 | Silicon oil | Perfluorocarbon Liquid residue |
| Fung NSK, et al. | ILM transposition and tuck technique | 821 μm | 0.33 | 87% | 8 | 20% SF6 | Difficult to tuck the curled, soft, and pliable ILM into a macular hole |
| Hu Z et al | Inverted ILM flap combined autologous blood clot technique | 754.4 μm | 0.63 | 96% | 25 | 15% C3F8 | Blood clot harvesting was troublesome and increased risk of infection |
| Grewal DS, et al | Autologous Neurosensory Retinal Free Flap transplantation | 1100 μm | 0.4 | 100% | 1 | Silicone oil | Retina harvesting may induce retinal detachment |
| San-Ni Chen | Capsular flap transplantation | 788.8 μm | 0.42~0.49 | 75% | 20 | 25% SF6 or 13% C3F8 | Lens capsule is difficult to be seen clearly without stained witch raise the concern of retinal toxicity. |
| Magno A Ferreira et al. | Human amniotic membrane plug transplant | 1302 μm | 0.3 | 100% | 19 | 20% SF6 or 14% C3F8 | Fresh hAM plug was not available in many hospital |
| Our study | ILM flap inverting under air | 683.0 μm | 0.56 | 100% | 10 | Air | Short learning curve |

ILM: Internal limiting membrane; MH: macular hole.
